# Computational Modeling of Motile Cilia-Driven Cerebrospinal Flow in the Brain Ventricles of Zebrafish Embryo

**DOI:** 10.3390/bioengineering9090421

**Published:** 2022-08-28

**Authors:** Huseyin Enes Salman, Nathalie Jurisch-Yaksi, Huseyin Cagatay Yalcin

**Affiliations:** 1 Department of Mechanical Engineering, TOBB University of Economics and Technology, Ankara 06510, Turkey; 2 Department of Clinical and Molecular Medicine, Faculty of Medicine and Health Sciences, Norwegian University of Science and Technology, 7491 Trondheim, Norway; 3 Biomedical Research Center, Qatar University, Doha, Qatar

**Keywords:** computational fluid dynamics, motile cilia, cerebrospinal flow, embryonic development, zebrafish, ANSYS, brain ventricles

## Abstract

Motile cilia are hair-like microscopic structures which generate directional flow to provide fluid transport in various biological processes. Ciliary beating is one of the sources of cerebrospinal flow (CSF) in brain ventricles. In this study, we investigated how the tilt angle, quantity, and phase relationship of cilia affect CSF flow patterns in the brain ventricles of zebrafish embryos. For this purpose, two-dimensional computational fluid dynamics (CFD) simulations are performed to determine the flow fields generated by the motile cilia. The cilia are modeled as thin membranes with prescribed motions. The cilia motions were obtained from a two-day post-fertilization zebrafish embryo previously imaged via light sheet fluorescence microscopy. We observed that the cilium angle significantly alters the generated flow velocity and mass flow rates. As the cilium angle gets closer to the wall, higher flow velocities are observed. Phase difference between two adjacent beating cilia also affects the flow field as the cilia with no phase difference produce significantly lower mass flow rates. In conclusion, our simulations revealed that the most efficient method for cilia-driven fluid transport relies on the alignment of multiple cilia beating with a phase difference, which is also observed in vivo in the developing zebrafish brain.

## 1. Introduction

Motile cilia are hair-like microscopic structures, which perform active periodic beating [1,2,3,4,5,6]. They are responsible for generating a directional flow to provide mixing and fluid transport for various biological processes [5,7,8,9]. In vertebrates, motile cilia are present in several organs, including the respiratory pathways, nervous system, left–right organizer, and oviducts [8,9,10,11,12,13,14,15]. The functions of motile cilia are numerous. These include mucus clearance in the respiratory system, breaking the left–right symmetry during embryonic development, and movement of cerebrospinal fluid (CSF) in the brain and spinal cord [8,16,17,18,19,20,21,22,23]. Hence, motile cilia defects can lead to a variety of symptoms in humans, including lung diseases, left–right asymmetry defects, and hydrocephalus [11,24,25].

There are a number of numerical studies investigating cilia-mediated transport in the respiratory system [5,26,27,28,29,30], the left–right organizer [31,32,33,34], and to a lesser extent in the brain [35,36]. In the brain, motile cilia of ependymal cells generate a directional flow of CSF along the brain ventricles [8,22,23,37], whereas the blood circulation, respiration, glymphatic system, and CSF secretion contribute to the bulk flow of CSF throughout the nervous system [10,38,39,40,41,42]. Given the limited number of numerical investigations, the exact mechanism of cilia-generated CSF flow in the brain ventricles remains elusive [8,9,43]. This in turn limits our understanding of how ciliary beating, in contrast to bulk flow, supports the various functions of CSF, which include the transport of nutrients and signaling molecules, the hydromechanical protection of the brain, and the removal of waste [44,45,46].

Motile cilia come in different forms depending on the organ and development stage analyzed [5,10,22,47]. Cilia may either be solitary or are organized in brush on multi-ciliated cells [47,48]. Their ciliary waveform can also either be rotational or whip-like in the case of multi-ciliated cells [49,50]. Previous findings in the literature reveal that the beating frequency, cilia length, beating pattern, and cilia density have important effects on the generated cilia-driven flow [4,5,28,30,51,52,53]. It is therefore not surprising that ciliary defects lead to a decreased forward flow rate [51] and organ dysfunction [11,24,25]. A complex flow field can be generated during cilia motion with the appearance of small-sized vortices on the cilia tips [28,51]. It is also reported that the increasing cilia length provides an increased directional flow rate, and a smaller cilia length with a random beating pattern results in increased flow mixing [54]. Besides, the way ciliary beating is synchronized and how synchronization modulates fluid pumping remain poorly understood [55]. Notably, metachronal waves, which refer to cilia beating with a time delay between neighbors [1,56], have been commonly described for multi-ciliated cells and are suggested to improve fluid pumping [55,57,58,59,60,61].

For a deeper understanding of the cilia-driven flow, it is necessary to numerically model the problem and solve the physically governing fluid flow equations during the movement of the cilia. Computational fluid dynamics (CFD) is an important modality for investigating the biological flows in the human body which cannot be directly analyzed using experimental visualization techniques, such as in cilia-driven flow [62,63]. The complex flow geometries can be extracted with the use of image-processing techniques [64]. Then, the generated geometries are isolated and physically relevant boundary conditions are applied to elucidate the flow patterns in the volume of interest [65]. The boundary conditions can be determined by measuring the instantaneous inflow velocities by utilizing visualization techniques such as computed tomography [66] or Doppler ultrasonography [67].

Zebrafish is a unique animal model for studying cilia-driven flows because of the transparency and small size of the zebrafish embryo, the availability of fluorescent lines to image beating cilia via microscopy, and the conservation of cilia-related processes [8,10,22,68,69]. Nevertheless, in vivo characterization of CSF flow, even in zebrafish embryo, is a challenging task due to the limitations in measuring the flow velocities at a microscopic scale. CFD modeling enables to generate cilia-driven flow fields by employing numerical techniques. The flow is mainly generated due to the interactions between the cilia and fluid particles in the flow domain. In the literature, there are several studies monitoring CSF movements in zebrafish embryos employing photoactivatable proteins or particles to determine CSF flow patterns [8,37,68,70,71]. These flow measurements aim to demystify the CSF fluid properties in embryonic zebrafish brain [72] and depend on in vivo investigations paired with advanced imaging techniques, such as optical coherence tomography [8,37]. However, there is a lack of studies that directly perform CFD analysis to simulate the cilia-driven CSF flow in embryonic zebrafish brains.

In this study, we aimed to estimate cilia-driven flow fields using experimentally measured movements of a solitary cilium by employing two-dimensional CFD simulations. The wall of the cilia is defined as a deforming solid wall which interacts with the surrounding fluid domain. The effects of cilia tilt angle, single and multiple cilia formations, and phase difference of beating patterns in time are investigated. The CFD models and applied modeling methodology are explained in Section 2. The results of computational simulations are presented in Section 3. Finally, the discussion of the findings and the limitations of the study are explained in Section 4. The findings of the computational flow models provided insight about the cilia-generated directional flow field in the brain ventricles of zebrafish embryos.

## 2. Materials and Methods

CFD modeling was performed using the commercial finite element software package ANSYS Workbench 19.2 (Canonsburg, PA, USA). For the preparation of the model geometry, ANSYS DesignModeler package was employed. The meshes and flow models were created, solved, and post-processed using ANSYS Fluent package.

In the literature, both 2D and 3D CFD models are employed to estimate the flow due to the cilia motion [51,73,74]. In our study, the cilia were modeled as membranes with indiscernible thickness. The cyclic motion of a cilium in the brain ventricle of a zebrafish embryo used in this study was experimentally determined as reported in a previously published study [8]. The determined motion was applied as a pre-defined boundary condition of a moving cilium model to investigate the cilia-driven flow. Since the dataset employed for the cilia movements provides a two-dimensional projection of in vivo measurements, we performed 2D CFD analysis to reveal the CSF flow.

### 2.1. Determination of Cyclic Cilia Motion

**Figure 1:**
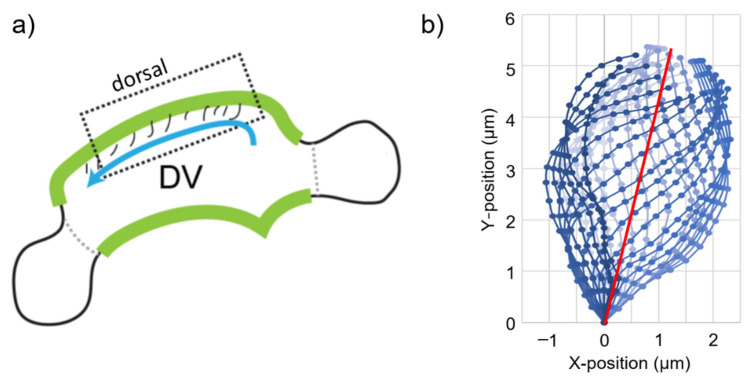
(**a**) The schematic of a brain ventricle for a 2 dpf zebrafish embryo. The blue arrow shows the flow direction. DV: Di-/mesencephalic ventricle. The green highlight indicates the location of motile cilia. (**b**) The averaged cyclic motion of one motile cilium. The color of the lines from light to dark reflects the progression of time in the cyclic motion. The red line shows the spatial mean of the cyclic motion and has an angle of 30 degrees with the Y-axis. The total time length of one cyclic motion was 0.033 s.

The 2D cilia waveform was obtained from a prior study where it was imaged in the dorsal di-/mesencephalic ventricle of a developing zebrafish brain ventricle at two days post-fertilization (dpf) [8] using a transgenic line expressing a fusion protein between a ciliary protein (Arl13b) and green fluorescent protein (Tg(beta-actin:arl13b-gfp) [75]). The waveform was recovered using the spermQ software (v0.2.2, Institute of Innate Immunity, Bonn, Germany) [76] and was used to determine the sample cyclic motions in two dimensions. In Figure 1a, the geometric form of a 2 dpf zebrafish brain ventricle is presented. The determined cyclic motion of motile cilia is shown in Figure 1b, where the red line shows the spatial mean line of the cyclic motion. The length of the cilia was 4.75 µm. The duration of a one full cyclic motion was 0.033 s, which corresponds to a beating frequency of approximately 30 Hz.

### 2.2. Model Geometry of CFD Models

**Figure 2:**
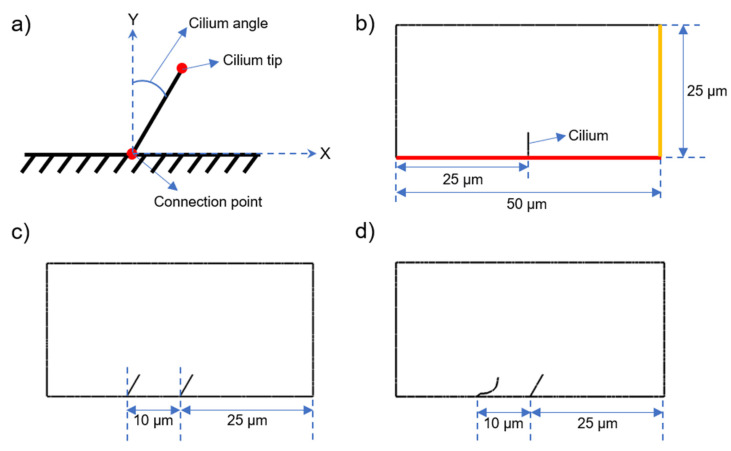
(**a**) Definition of cilium tilt angle. The cilia are connected to the ground and the connection point is stationary during the ciliary beating. (**b**) The dimensions of the flow domain are used as 25 µm × 50 µm. The red line is the ground of the flow domain, which is modeled as a wall with no-slip condition. A non-zero mass flow rate is observed on the yellow line depending on the ciliary motion. (**c**) The geometric configuration of two cilia with no phase difference (Case 4, in phase). (**d**) The geometric configuration of two cilia with phase difference (Case 5, out of phase). For the out of phase configuration, the cilium at the right side completes its cycle, while the cilium at the left is at half of the cyclic motion.

Five different cases were modeled in CFD simulations to reveal the effects of cilia tilt angle, multiple cilia, and phase difference in ciliary motion. As presented in Figure 2, the cilium tilt angle is defined as the angle between the Y-axis and the spatial mean line of the cyclic motion. In the first modeled case, only one cilium with a 30° cilium tilt angle was considered. In the second and third cases, one cilium was modeled separately with 50° and 60° cilium tilt angles. In these three cases, only a single cilium was modeled with different cilium tilt angles. In the fourth case, two neighboring cilia were modeled without any phase difference in the cyclic motion. Since there was no phase difference in the fourth case, the cilia moved in a synchronous way, which is called ‘in phase’. In the fifth model, two neighboring cilia were modeled with a phase difference, which is called ‘out of phase’. In the out of phase cilia movements, one of the cilia was at the end of the cyclic motion, while the other cilium was at the middle of the cyclic motion. In the fourth and fifth models, the effects of multiple cilia formation and phase difference were mainly investigated on the cilia-generated flow. Since the phase difference is defined between two moving cilia, the phase cannot be defined for a single cilium, which was employed in the first three modeled cases. Table 1 summarizes the parameters of the five different cases.

The parameters determined in Table 1 were selected to clearly reveal the effects of cilia quantity, cilia tilt angle, and beating phase of the cilia. The maximum tilt angle was set as 60° because the tilt angles greater than 60° cause a collision between the cilia and the ground. The minimum tilt angle was defined as 30°, because at a tilt angle of 30°, the spatial mean of the cilia movements becomes parallel to the Y-axis. Since the highest flow rates are expected at a cilia tilt angle of 60°, the phase comparison was performed considering the case of a 60° tilt angle.

As previously stated, a defect in cilia function can impair the forward CSF flow and consequently lead to neurological disorders [24,25]. Therefore, it is important to understand the role and contribution of cilia-related parameters such as tilt angle, quantity of cilia, and phase difference. For a more realistic flow analysis, the quantity of cilia should be increased in the flow domain. However, due to the limited computational power, one or two cilia were modeled in the CFD analysis.

The cilia were modeled as membranes with zero thickness and connected to the ground at one connection point. The connected point was fixed and did not move during the cyclic motion of the cilia. In CFD simulations, the prescribed cilia motion shown in Figure 1b was defined as moving boundary conditions on the membranes. In other words, the cilia were not modeled as structures and only modeled as moving boundaries. Since the cilia were not solid structures, there was no need to employ a constitutive equation for modeling the cilia material properties.

The flow domain geometry of the modeled cases is shown in Figure 2. The dimensions of the flow domain were modeled as 25 µm × 50 µm. The bottom line of the flow domain was considered as a wall with no-slip condition, which guaranteed that the flow velocity was zero on the ground. The other three bounding lines of the flow domain were set as free surfaces with zero pressure. As the motile cilia moved with the pre-defined cyclic motion, a flow field was generated in the fluid domain and a non-zero mass flow rate was observed on the right-side line of the flow domain, which is shown in yellow in Figure 2.

### 2.3. CFD Models

The flow fields were determined by solving the physically governing continuity and Navier–Stokes equations presented in Equations (1) and (2), respectively. In these equations, v defines the velocity vector in m/s, ρ denotes the mass density of the cerebrospinal fluid in kg/m^3^, and τ denotes the tensor of fluid stress in N/m^2^. The effect of gravity is neglected in the solution of CFD models due to the indiscernible effect on the cilia-generated flow velocities.

(1) ∇·v=0

(2) $$\rho \frac{\partial v}{\partial t}+\rho \left(v\right)\cdot \nabla v-\nabla \cdot \tau =0$$

The problem domain was discretized into a finite number of triangular elements to form a nonuniform mesh for solving the governing equations at each element. The size of each triangular mesh element was set around 0.25 µm. The total number of elements was approximately 42,000 for the generated meshes.

The laminar flow solver was employed due to the significantly low velocities expected in the flow domain. Each cilium was divided into 19 equal pieces and 20 nodes. During the cyclic movements, the positions of each cilium node were determined using the motion profile provided in Figure 1b. Ten consecutive ciliary beatings were simulated for each computational case, which resulted in a total time length of 0.33 s. Each cyclic ciliary beating was divided into 33 equal timesteps with 0.001 s increments. In total, 330 timesteps were solved for each different case to simulate 10 sequential ciliary beatings.

The cerebrospinal fluid was modeled as a Newtonian fluid using a mass density of 1000 kg/m^3^ and viscosity of 1.0 centipoise (cP) [77,78]. In Newtonian fluids, the fluid viscosity was constant during the analysis, regardless of the flow conditions. An incompressible fluid assumption was used in the CFD analysis because the material properties of CSF are nearly identical to those of water. Since the flow was generated by a cyclic motion of the motile cilium, there was no assigned Reynolds number in the flow domain and the Reynolds number locally changed according to the position of the fluid particle in the flow domain.

## 3. Results

**Figure 3:**
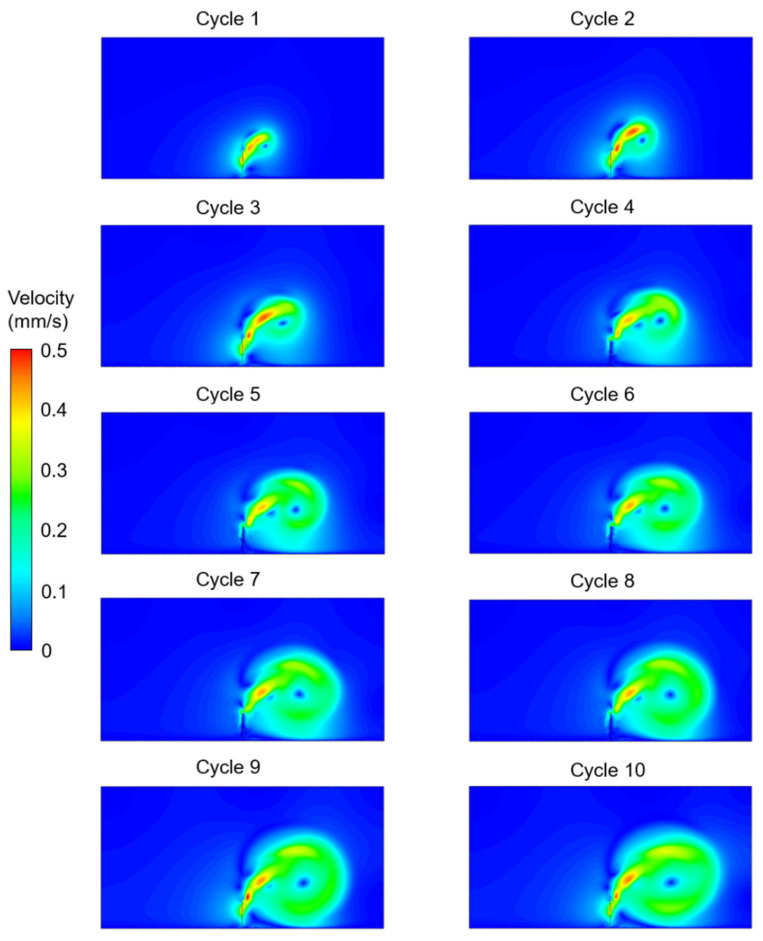
Velocity contour plots of Case 1 for a single cilium with a 30° cilium tilt angle. The flow fields are provided at the end of each ciliary cycle.

**Figure 4:**
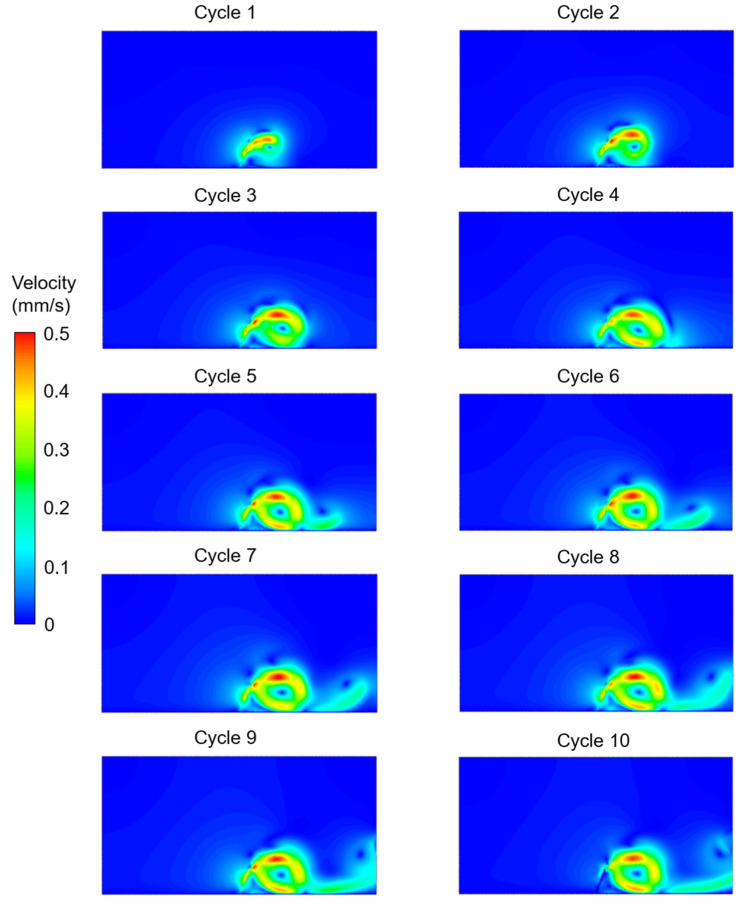
Velocity contour plots of Case 2 for a single cilium with a 50° cilium tilt angle. The flow fields are provided at the end of each ciliary cycle.

**Figure 5:**
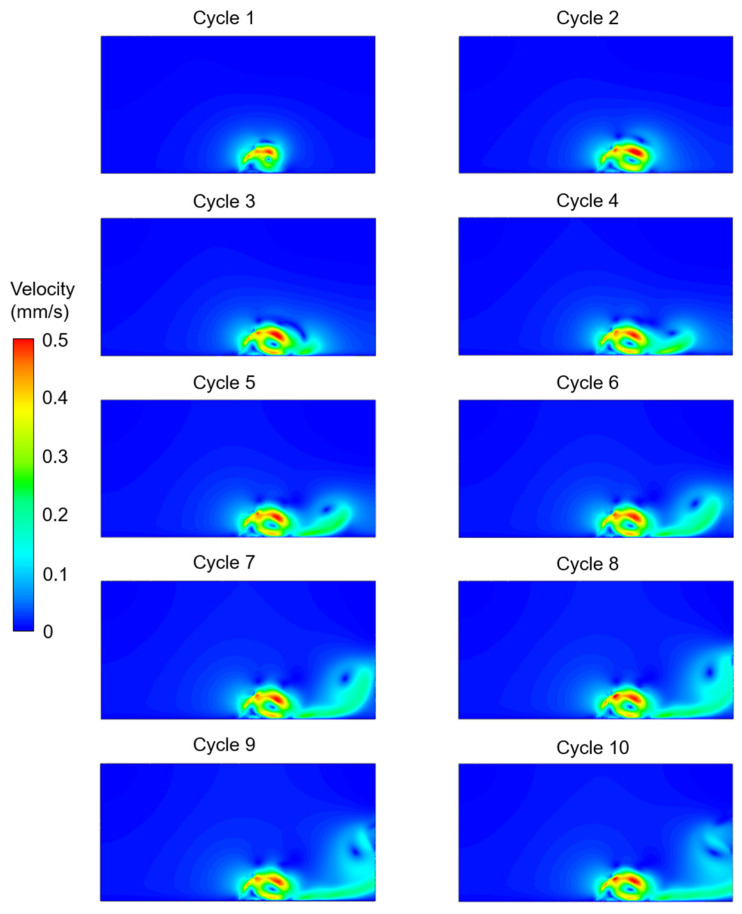
Velocity contour plots of Case 3 for a single cilium with a 60° cilium tilt angle. The flow fields are provided at the end of each ciliary cycle.

The velocity contours were determined for the five different investigated cases considering the effects of cilium tilt angle, quantity of cilia (1 or 2), and phase difference between the beating cilia. In the first three cases, only one single cilium was modeled with different cilium angles. In Figure 3, Figure 4 and Figure 5, the velocity contour plots of Case 1, Case 2, and Case 3 are provided for cilium angles of 30°, 50°, and 60°, respectively. In the figures, the velocity contours are determined at the end of each ciliary cycle. The same color-coded velocity scales are used for all presented results for ease of comparison.

For a 30° cilium tilt angle, the ciliary beating resulted in a recirculating vortex which was growing with the increasing time. The direction of flow was observed to be from left to right due to the asymmetrical motion of the motile cilium. When the cilium tilt angle increased to 50°, a directional flow was observed after the fourth ciliary beating. For a 50° cilium tilt angle, the directional flow had a maximum velocity of approximately 0.225 mm/s at the end of the tenth ciliary cycle. When the cilium tilt angle was increased to 30°, the generated flow field was quite similar to the result of the 50° cilium angle in terms of the general flow behavior. For the cilium tilt angle of 30°, the directional flow velocity was increased to approximately 0.25 mm/s at the end of the tenth cycle.

**Figure 6:**
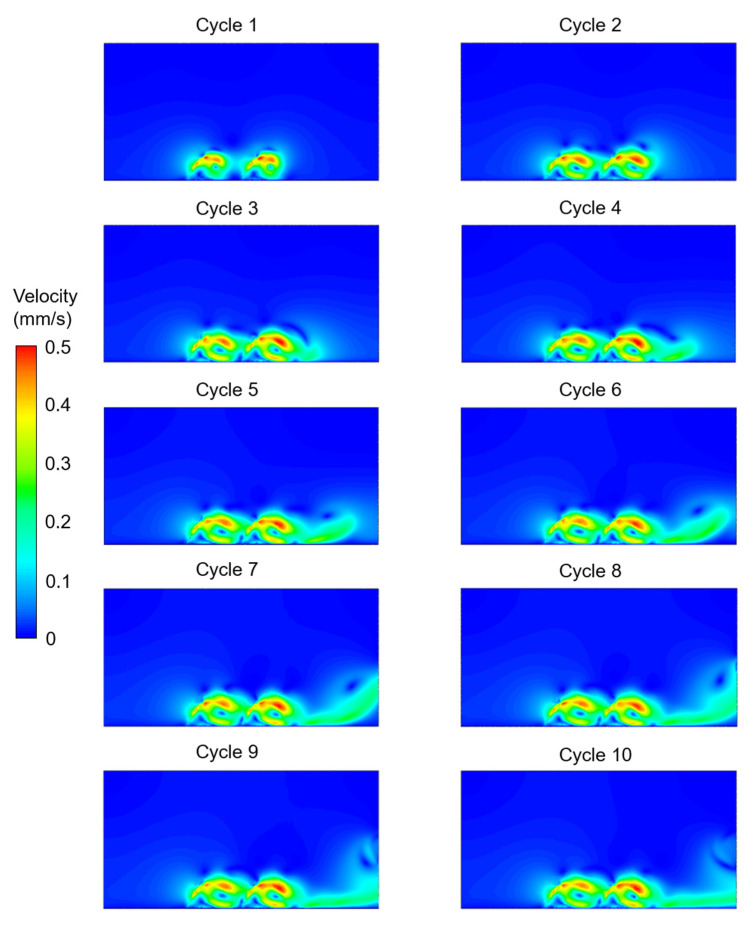
Velocity contour plots of Case 4 for two cilia with a 60° cilium tilt angle. The cilia beat synchronously (in phase) without any phase difference in time. The flow fields are provided at the end of each ciliary cycle.

**Figure 7:**
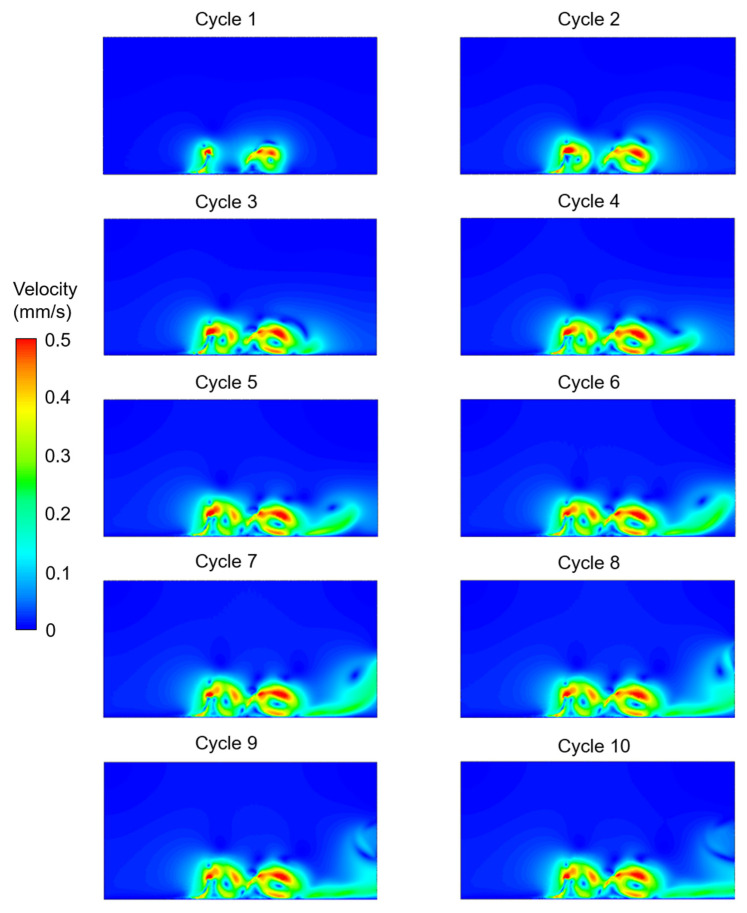
Velocity contour plots of Case 5 for two cilia with a 60° cilium tilt angle. The cilia beat with a phase difference (out of phase). One of the cilia completes the cyclic motion when the other one is at half of the cyclic movement. The flow fields are provided at the end of each ciliary cycle.

In Figure 6, the effect of multiple cilia with no phase difference (Case 4) was investigated. Compared to a single cilium with a 60° cilium tilt angle (Case 3), it was observed that the motion of the two cilia in phase (Case 4) resulted in a secondary vortex. For the two cilia configurations in Case 4 and Case 5, two recirculating vortices were observed in the flow domain. One of the vortices was between the two neighboring cilia, and the other vortex was at the downstream of the directional flow. In Figure 7, multiple cilia with a phase difference in ciliary beatings (Case 5) were investigated to reveal the consequences of a time delay in the cyclic movements of adjacent cilia. Among the five investigated cases, the generated directional flow of Case 5 had the highest velocity, which was around 0.3 mm/s. According to the CFD findings, all cases except Case 1 resulted in a directional flow toward the right side. Only in Case 1, with a cilium tilt angle of 30°, a large rotating vortex was observed instead of a directional flow.

**Figure 8:**
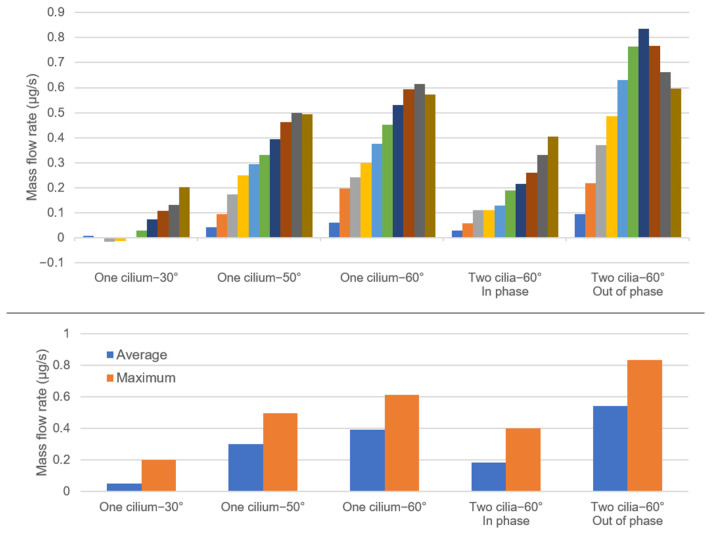
Mass flow rates at the right-side line of the flow domain for various tilt angles and cilia formations. The average mass flow rates are shown in the top figure, which uses ten different colors to represent ten consecutive cyclic ciliary beatings. In the bottom figure, the average and maximum mass flow rates during ten consecutive ciliary beatings are presented.

The amount of mass flow rate was determined on the right-side line of the flow domain at each cycle and presented in Figure 8. For Case 1 (one cilium, 30° cilium tilt angle), a backflow was observed at the third and fourth cycles. For cases 2, 3, 4, and 5, there was no backflow at any instant during the ten cycles. The average mass flow rates during ten ciliary beating cycles were determined as approximately 0.05, 0.30, 0.39, 0.18, and 0.54 µg/s, for the cases 1, 2, 3, 4, and 5, respectively. The maximum mass flow rates during the ten investigated cycles were determined as approximately 0.20, 0.49, 0.61, 0.40, and 0.84 µg/s, for the cases 1, 2, 3, 4, and 5, respectively. The Case 5 with out-of-phase ciliary motion had the highest magnitudes in terms of both the average and maximum mass flow rates.

## 4. Discussion

The comparison of five different cases revealed that the cilium tilt angle, quantity of cilia, and phase difference in the cilia movements impacted the generated cilia-driven flow. In the first three cases, the effect of cilium tilt angle was elucidated, and it was observed that the 30° cilium tilt angle significantly reduced the amount of generated mass flow rate toward the right side. When the cilium tilt angle was increased, a directional flow was clearly observed and the amount of mass flow rate gradually increased [79]. When the cilium tilt angle was increased from 30° to 50°, a 6-fold increase was observed in the average mass flow rate and a 2.5-fold increase was observed in the maximum mass flow rate. When the cilium tilt angle was increased from 50° to 60°, the average mass flow rate increased by 30% and the maximum mass flow rate increased by 23%. A positive correlation was determined between the cilium tilt angle and the generated mass flow rate, indicating that the higher cilium tilt angles result in a stronger directional flow. As the cilium angle increased, the generated vortex hit the fixed ground and the directional flow separated from the recirculating flow. Therefore, if the ciliary beating pattern is close to the ground, a higher flow velocity is expected depending on the cyclic motion.

In Case 3 and Case 4, we investigated the impact of an increased cilia quantity on flow rates with a cilium tilt angle set to 60°. Interestingly, we observed that two cilia beating in-phase reduced the average and maximum mass flow rates as compared to a single cilium. Notably, there was a 53% decrease in the average mass flow rate, and a 34% decrease in the maximum mass flow rate. This reduction is considered to be related to the synchronous motion of the adjacent cilia [80]. In a synchronous motion, the cilia follow each other like a shadow and the flow force generated by one cilium can be damped and blocked by the other one, resulting in a reduced flow force [1,81].

In the comparison of Case 4 and Case 5, we then investigated the effect of phase difference. In this scenario, we observed that the phase difference between the two neighboring cilia significantly increased the average and maximum mass flow rates. Case 5 with out-of-phase beating led to an approximately 3-fold increase in the average mass flow rate and a 2-fold increase in the maximum mass flow rate when compared to Case 4 with no phase difference. In Case 5, the maximum flow rate was observed at the end of the sixth cycle, and the amount of flow rate decreased up to the tenth cycle. A similar behavior was also observed in the results of Case 2 and Case 3, where the maximum flow rate was reached at the end of the ninth cycle. This suggests that the flow rate reaches a maximum level before it scales down eventually. In addition to the increased directional flow velocity, the phase difference also resulted in enhanced mixing in the flow domain due to the variations in the mass flow rates during ciliary beating [80]. Therefore, the most effective case is considered to be Case 5, with multiple cilia beating out of phase, which is also observed in the physiological ciliary beatings in the brain ventricle of zebrafish embryos [1,8]. Hence, our results suggest that the high levels of mass flow rate enable improved particle transport and fluid mixing within the brain ventricles [25,81].

In the five different modeled cases, the maximum flow velocity was obtained in the order of 100 µm/s. The in vivo flow measurements revealed that time-averaged flow velocity in 2 dpf zebrafish brain ventricle was around 10 µm/s [8,68]. However, in these measurements, the time and spatial resolutions were limited and the velocities were averaged over large domains of the ventricles, which resulted in underestimated average flow velocities [8]. From this perspective, the maximum flow velocities determined in the current investigation are physiologically consistent with the in vivo flow conditions. For a more accurate numerical analysis, nearly 80 cilia should be modeled on the dorsal wall considering different beating frequencies and waveforms. Determination of 3D ciliary beating profiles and solving 3D CFD models would improve the solution accuracy of the investigated flow domain. Due to the limited computational power, the solutions of CFD simulations were determined up to the tenth ciliary beating cycle. In further investigations, the quantity of cilia and the total number of investigated beatings are aimed to be increased. Nevertheless, this study provided important conceptual results to reveal the effect of cilia tilt angle and phase difference in the brain ventricles during the zebrafish embryonic development stage when cells bear a solitary motile cilium.

## 5. Conclusions

In this study, we applied CFD simulations to examine the flow produced by motile cilia. A 2D flow model was used to examine the effects of cilium tilt angle, multiple cilia formation, and phase difference in cilia movements. Overall, we observed a directional flow in all modeled cases due to the cilia’s asymmetrical cyclic movements. For the cilium tilt angle of 30°, cilia motion only produced a rotational vortex in the flow domain. However, as the cilia became closer to the ground with an increased tilt angle, we observed an enhanced directional flow. Interestingly, we identified that if two adjacent cilia moved without any phase difference, the mass flow rates were reduced in the flow domain. On the other hand, consecutive cilia beating with a phase difference in time significantly increased the flow velocity and mass flow rate, demonstrating that this is the most effective approach to transport the fluid particles in this simplified view of the embryonic zebrafish brain ventricles.

## Figures and Tables

**Table 1:** The modeled cases in CFD simulations.

	Quantity of Cilia	Cilium Tilt Angle	Phase between the Cilia
Case 1	1	30°	
Case 2	1	50°	
Case 3	1	60°	
Case 4	2	60°	In phase
Case 5	2	60°	Out of phase

## Data Availability

Not applicable.
